# Predictors of health-related quality of life in patients with knee osteoarthritis

**DOI:** 10.1186/s12891-026-10306-0

**Published:** 2026-08-01

**Authors:** Emrah Afsar, Zeynep Sena Namaz

**Affiliations:** 1 Department of Occupational Therapy, Faculty of Health Sciences, Kutahya Health Sciences University, Germiyan Campus, Kutahya, 43020 Turkey; 2 Therapy and Rehabilitation Department, Occupational Therapy Programme, Kutahya Health Sciences University, Tavsanli Health Services Vocational School, Kutahya, Turkey

**Keywords:** Osteoarthritis, Health related quality of life, Symptoms, Functional status, Depressive symptoms, Structural equation modeling

## Abstract

**Background:**

Identifying the predictors of health-related quality of life (HRQoL) is essential for improving patient outcomes in chronic conditions like knee osteoarthritis (KOA). However, research exploring the structural pathways within this context remains limited. This study aims to examine the structural relationships of symptoms, functional status, and depressive symptoms on both physical and mental HRQoL using Structural Equation Modeling (SEM).

**Methods:**

This study included 174 patients with KOA. For the assessment of patients, symptoms were measured by Numeric Pain Rating Scale, Western Ontario and McMaster Universities Osteoarthritis Index (WOMAC) (pain and stiffness); functional status by the Lower Extremity Functional Scale and WOMAC physical function; depressive symptoms by the Beck Depression Inventory. HRQoL physical and mental component were assessed using the Short-Form Health Survey (SF-36). SEM was used to examine the structural relationships of symptoms, functional status and depressive symptoms on HRQoL (physical and mental).

**Results:**

The final model exhibited the following fit indices (x^2^/df = 2.254, CFI = 0.941, IFI = 0.942, NNFI (TLI) = 0.918, RMSEA = 0.085, SRMR = 0.0802). This study revealed that symptoms were significantly associated with functional status (standardized coefficient = 0.978; *p* < 0.001), functional status with depressive symptoms (0.377; *p* < 0.001), and depressive symptoms with HRQoL-Mental (-0.773; *p* < 0.001). The findings showed that HRQoL-Mental was significantly associated with HRQoL-Physical (0.746; *p* < 0.05). However, neither symptoms nor functional status demonstrated a statistically significant association with HRQoL-Mental. Additionally, symptoms, functional status, and depressive symptoms showed no significant associations with HRQoL-Physical.

**Conclusions:**

The results of this study demonstrate the pathways associated with HRQoL in patients with KOA. Through a sequential pathway, symptoms and functional impairments are associated with higher depressive symptoms and lower HRQoL-Mental. This subsequent decline in HRQoL-Mental is associated with HRQoL-Physical. From a clinical perspective, this mediating relationship should be considered when patients show no improvement in their quality of life despite conventional or surgical treatments. Ultimately, to achieve a comprehensive improvement in quality of life in patients with KOA, it is essential to integrate symptom relief, the management of functional impairment, and the treatment of accompanying depressive symptoms into a holistic treatment plan.

**Trial registration:**

The study was registered at ClinicalTrials.gov on January 6, 2025 (Registration number: NCT06760338).

## Background

Osteoarthritis is a common condition that leads to disability and places a significant burden on affected individuals, healthcare systems, and socioeconomic costs [1]. Examining the anatomical distribution of this prevalent joint pathology reveals that knee osteoarthritis constitutes the subgroup with the highest rates of morbidity and disability [2, 3]. The seriousness of the disability burden is further highlighted by the prevalence of the disease. Recent global epidemiological data show that the prevalence of knee osteoarthritis (KOA) in individuals aged 40 and older is approximately 22.9%, and the global incidence rate is an average of 203 new cases per 10,000 person-years [3].

The most common symptoms associated with KOA are pain and stiffness [4, 5]. Structural changes in KOA cause chronic mechanical pain [4]. Patients naturally restrict their range of motion and use strategies to compensate for this, which alters joint kinematics [6]. Ultimately, this leads to periarticular muscle weakness [7], increased biomechanical stress and a vicious cycle of escalating pain and loss of physical function [8]. The most concrete and limiting reflection of this gradual decline in physical capacity is the loss of independence in an individual’s daily living activities. Activities that directly challenge the load-bearing capacity of the lower extremities, such as walking, climbing stairs, rising from a chair, or bending over, can result in functional limitations for individuals with KOA [9, 10]. The progressive decline in mobility associated with these functional limitations may contribute to psychological distress. When combined with the persistent symptoms of KOA, including pain and stiffness, this decline can impose a substantial psychological burden on affected individuals. This physical functional impairment in patients with KOA has been demonstrated to be a critical factor in the onset of depressive symptoms [10, 11]. As demonstrated in previous studies, the prevalence of depressive symptoms within this population ranges from 19.9% to 25.4% [12, 13].

KOA is not a life-threatening disease; however, it is associated with a diminished quality of life and reduced levels of physical, mental and social well-being [14]. According to the World Health Organisation definition, quality of life is “an individual’s perception of their position in life in the context of the culture and value systems in which they live and in relation to their goals, expectations, standards and concerns” [15]. Health-related quality of life (HRQoL) represents the aspect of overall quality of life specifically attributable to an individual’s health [16, 17]. Previous studies have demonstrated that patients with knee osteoarthritis exhibit significantly lower HRQoL profiles compared to healthy controls [18, 19]. In the literature, studies have shown that the HRQoL of patients with KOA is affected by various factors, including sociodemographic characteristics, pain intensity, functional status of the lower extremities, and depressive symptoms [20–23]. Furthermore, the existing literature highlights that identifying the specific physical and psychological determinants of HRQoL is crucial for improving patient prognosis and facilitating effective rehabilitation [24]. While previous studies have evaluated these factors, integrated models that simultaneously examine their interactions and map them to distinct physical and mental dimensions of HRQoL are inadequate. Moreover, the complex structural relationships among these factors and their explanatory role in HRQoL remain insufficiently investigated. The present study aims to utilise Structural Equation Modeling (SEM) to determine the pathways between these variables, and to evaluate their structural relationships and overall association with the HRQoL in patients with KOA.

## Methods

### Study design

This study was planned as descriptive and cross-sectional. The study was approved by Kutahya Health Sciences University Non-Interventional Clinical Research Ethics Committee (2024/14–29) and registered at ClinicalTrials.gov on January 6, 2025 (Registration number: NCT06760338). The procedures were performed in accordance with the principles of the Declaration of Helsinki.

### Participants

The sample of this study consists of patients with knee osteoarthritis. Participants were contacted through social media and personal contacts. A total of 174 patients with knee osteoarthritis who met the inclusion criteria were included in the study. The inclusion criteria were as follows: willing to participate in the study; diagnosis of knee osteoarthritis; and being aged between 40 and 65 years. Exclusion criteria were: history of orthopedic surgeries in the lower extremity, presence of neurological diseases, communication or cognitive deficits.

### Recruitment and setting

Participants were adults who had previously received a clinical diagnosis of KOA from a physician at a hospital or outpatient clinic. They were recruited using a snowball sampling approach. Initially, study invitations were disseminated through social media platforms, and healthcare professionals who encountered the invitation were encouraged to refer eligible patients to the study. Subsequently, these initial participants were invited to forward the study invitation to other eligible individuals. The participants reported having been previously diagnosed with KOA by a physician. Data collection was carried out at Kutahya Health Sciences University Disability Research and Application Centre between January – July 2025. All patients were informed about the procedures and provided written informed consent to enroll in the study. All assessments were administered in a single session for each participant to ensure consistency.

### Assessments

#### Patient demographic data

Data regarding the participants’ age, gender, height, weight, body mass index, and pain duration were collected using a data form.

#### Numeric pain rating scale (NPRS)

Pain intensity was assessed using the Numeric Pain Rating Scale (NPRS). Participants were asked to rate the severity of their pain from 0 points (no pain) to 10 points (worst pain imaginable) over the past 24 h [25]. The NPRS are commonly used pain measure in the clinical setting [26]. The test-retest reliability of the scale was found to be high (*r* = 0.82) in patients with chronic pain [27].

#### Western Ontario and McMaster universities osteoarthritis index (WOMAC)

Pain, stiffness and physical function level were assessed using the Western Ontario and McMaster Universities Osteoarthritis Index (WOMAC). The WOMAC questionnaire comprises 24 questions and three subscales (pain, stiffness and physical function). The subscales are as follows: pain (5 questions), stiffness (2 questions), and physical function (17 questions). Each question is scored on a scale of 0–4 (0 = absent, 1 = mild, 2 = moderate, 3 = severe, 4 = extreme). The maximum possible scores are 20 points for pain, 8 points for stiffness and 68 points for physical function. A high WOMAC score indicates worse pain, stiffness, and physical function [28]. The Turkish version of the WOMAC questionnaire has been shown to be a valid and reliable in patients with knee osteoarthritis [29].

#### Lower extremity functional scale (LEFS)

The Lower Extremity Functional Scale (LEFS) was used to assess lower extremity functional status. LEFS comprises a total of 20 items. Each item is scored between 0 (extreme difficulty) and 4 (no difficulty at all). The total score ranges from 0 to 80. Higher scores indicate a better functional condition [30]. The Turkish version of the LEFS questionnaire has been shown to be a valid and reliable [31].

#### Beck depression inventory (BDI)

The BDI was used to assess the depressive symptoms of participants. The BDI is a widely used tool for assessing the psychological state of individuals with chronic musculoskeletal disorders [32, 33]. The BDI is a 21-item questionnaire. Each item is scored from 0 (absent) to 3 (severe). The total BDI score varies between 0 and 63. A higher score indicates more severe depressive symptoms [34]. The Turkish version of the BDI has been shown to be valid and reliable [35].

#### RAND 36-Item short form health survey (SF-36)

The RAND 36-Item Short Form Health Survey (SF-36) was used to assess the HRQoL of the participants. SF-36 is a 36-item self-reported questionnaire consisting of 8 subscales: physical functioning, role limitations due to physical health, role limitations due to emotional problems, vitality (energy/fatigue), emotional well-being, social functioning, bodily pain and general health perceptions. These 8 subscales are scored between 0 (worst health state) and 100 (best health state). Higher scores indicate better health status [36]. The Turkish version of SF-36 has been proven to be valid and reliable [37].

### Statistical analysis

The data of the study were analyzed using IBM Statistical Package for Social Sciences (SPSS) 23.0 (SPSS Inc., Chicago, IL, USA) and Analysis of Moment Structures (AMOS) statistical package programs. In all analyses, *p* < 0.05 was considered statistically significant. No missing or incomplete data were observed for any of the variables included in the analysis. The univariate normality of the data was assessed using skewness and kurtosis values. Descriptive statistics for numerical variables were given as minimum-maximum (Min-Max), mean (Mean) and standard deviation (Sd). Categorical variables were presented as frequencies and percentages.

A SEM was constructed with AMOS software by selecting the maximum likelihood method in order to examine predictor effects of symptoms, functional status, depressive symptoms on HRQoL (physical and mental). Prior to testing the structural model, the potential for multicollinearity among the predictor variables was evaluated using Tolerance and Variance Inflation Factor (VIF) values. The following model fit indices were used: chi-square (x^2^) and degrees of freedom (df) a x^2^/df ratio between 0 and 2 indicates a perfect fit, and between 2 and 3 indicates an acceptable fit; Comparative Fit Index (CFI) values between 0.95 and 1.00 indicate a perfect fit, and between 0.90 and 0.95 an acceptable fit; Incremental Fit Index (IFI) values between 0.95 and 1.00 indicate a perfect fit, and between 0.90 and 0.95 an acceptable fit; Non- Normed Fit Index (NNFI, also known as the Tucker – Lewis Index, TLI) values between 0.95 and 1.00 indicate a perfect fit, and between 0.90 and 0.95 an acceptable fit [38, 39]. RMSEA values between 0 and 0.05 are considered a ‘good fit,’ values between 0.05 and 0.08 indicate an ‘adequate fit,’ and values between 0.08 and 0.10 are classified as a ‘mediocre fit.’ Values exceeding 0.10 are deemed ‘not acceptable’. For SRMR, values up to 0.05 represent good fit, while values between 0.05 and 0.10 are considered acceptable [40, 41].

## Results

A total of 174 patients with knee osteoarthritis were included in the study. Of the patients, 69.5% (*n* = 121) were female and 30.5% (*n* = 53) were male. Information about the age (year), BMI (kg/m^2^), and pain duration (months) values of the participants are summarized in Table 1. The descriptive statistics of the study variables are presented in Table 2.

**Table 1:** Demographic characteristics of the participants

	Min-Max (*N*:174)	x̄ ± Sd (*N*:174)
Age (years)	40–65	53.54 ± 7.13
BMI (kg/m^2^ )	18.20–44.90	29.22 **±** 4.31
Pain duration (months)	6-240	75.22 **±** 57.57

*BMI* body mass index, *kg/m*^2^ kilogram per square meter, *N* total number of participants, *Min* minimum value, *Max* maximum value, x̄ mean value, *Sd* standard deviation value

**Table 2:** Descriptive statistics of the study variables

Variables	Min-Max (*N*:174)	x̄ ± Sd (*N*:174)
Pain Intensity (NPRS) (0–10)	4–9	7.44 **±** 1.08
WOMAC Pain Score (0–20)	5–20	15.79 **±** 2.81
WOMAC Stiffness Score (Range: 0–8)	0–8	5.86 **±** 1.84
WOMAC Physical Function Score (0–68)	26–68	51.87 **±** 9.67
Lower Extremity Functional Scale (LEFS) (0–80)	0–66	22.55 **±** 11.76
Beck Depression Inventory (BDI) (0–63)	0–37	10.45 **±** 6.29
SF-36 Physical Functioning (0–100)	0–90	46.61 **±** 18.32
SF-36 Role Limitations due to Physical Health (0–100)	0-100	1.15 **±** 10.70
SF-36 Role Limitations due to Emotional Problems (0–100)	0-100	76.82 **±** 40.94
SF-36 Vitality (Energy/Fatigue) (0–100)	10–80	48.48 **±** 12.40
SF-36 Emotional Well-Being (0–100)	12–84	61.43 **±** 13.13
SF-36 Social Functioning (0–100)	12.50–100	55.39 **±** 17.08
SF-36 Bodily Pain (0–100)	0-67.5	35.52 **±** 13.72
SF-36 General Health Perceptions (0–100)	10–70	43.19 **±** 14.23

*WOMAC* Western Ontario and McMaster Universities Osteoarthritis Index, *SF-36* RAND 36-Item Short Form Health Survey, *BMI* body mass index, *kg/m*^2^ kilogram per square meter, *N* total number of participants, *Min* minimum value, *Max* maximum value, x̄ mean value, *Sd* standard deviation value

The univariate normality of the data was assessed using skewness and kurtosis values. The skewness and kurtosis values for the study variables remained within acceptable ranges of ± 3 and ± 10, respectively, indicating that the normality assumption was satisfied [42, 43].

Prior to testing the structural model, the potential for multicollinearity among the predictor variables was evaluated using Tolerance and Variance Inflation Factor (VIF) values. The analysis revealed that all Tolerance values exceeded 0.10, and all VIF values were below 10. These findings confirm the absence of multicollinearity issues among the independent variables, satisfying the assumptions recommended in the literature [44, 45].

**Fig. 1:**
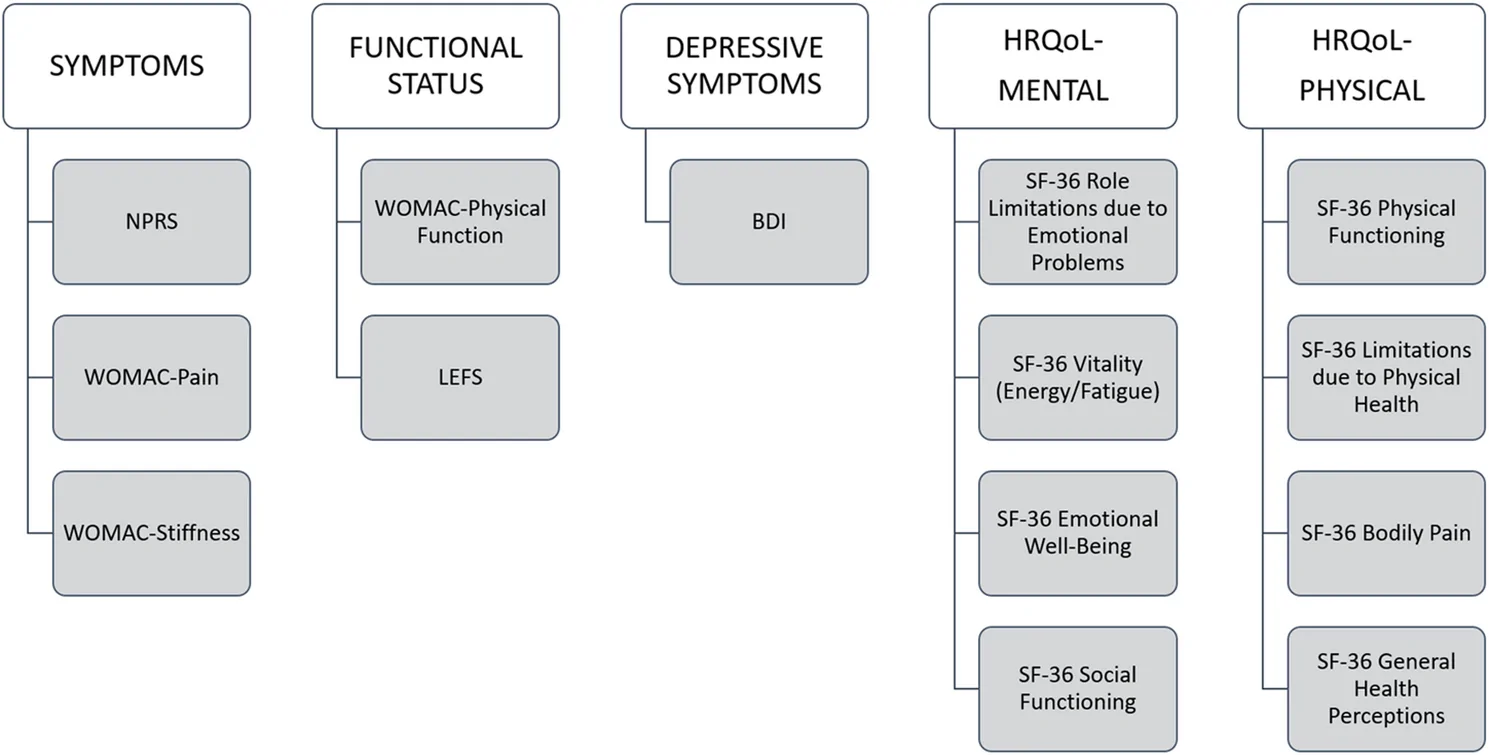
Outcome measures. BDI, Beck Depression Inventory; HRQoL_MENTAL, Health-Related Quality of Life Mental Component; HRQoL_PHYSICAL, Health-Related Quality of Life Physical Component; LEFS, Lower Extremity Functional Scale; NPRS, Numeric Pain Rating Scale; SF-36, RAND 36-Item Short Form Health Survey; WOMAC-Pain, Western Ontario and McMaster Universities Osteoarthritis Index - Pain; WOMAC-Physical Function, Western Ontario and McMaster Universities Osteoarthritis Index - Physical Function; WOMAC**-**Stiffness, Western Ontario and McMaster Universities Osteoarthritis Index – Stiffness

During the model development phase, scales and sub-scales scores that were theoretically related were combined. Accordingly, symptoms, functional status, depressive symptoms, and HRQoL (physical and mental) structures were included in the model as latent variables represented by the relevant scale scores. The NPRS, WOMAC-Pain, and WOMAC-Stiffness were specified as indicators of the symptoms, as they capture the primary joint complaints of osteoarthritis. The WOMAC-Physical Function and the LEFS were assigned to functional status because they directly measure activity limitations and lower-extremity mobility restrictions. Depressive symptoms were represented as a distinct construct using the BDI. Furthermore, HRQoL was divided into physical and mental constructs based on the alternative scoring method derived for the SF-36 [46]. Following this theoretical basis, four subscales (Physical Functioning, Role Limitations due to Physical Health, Bodily Pain, and General Health) formed HRQoL-Physical, while the remaining four subscales (Emotional Well-Being, Role Limitations due to Emotional Problems, Social Functioning, and Vitality) formed HRQoL-Mental. Thus, the measurement model was constructed based on the intended clinical definitions of the constructs (Fig. 1). The relationships between symptoms, functional status, depressive symptoms, and HRQoL in patients with KOA were examined using SEM. Observed variables are indicated by squares; latent variables (measured by multiple indicators) are indicated by ellipses.

The structural model was initially tested to verify the hypothesized relationships among symptoms, functional status, depressive symptoms, and HRQoL. Modifications were implemented based on both Modification Indices and theoretical justifications. To improve model fit, Modification Indices were examined, and theoretically justifiable covariances were added between error terms. Specifically, adding error covariances that were located under the same latent variable and provided a significant contribution to the model fit was preferred. The final model exhibited the following fit indices (x^2^/df = 2.254, CFI = 0.941, IFI = 0.942, NNFI (TLI) = 0.918, RMSEA = 0.085, SRMR = 0.0802). These findings demonstrate that the model structure is consistent with the established criteria.

**Fig. 2:**
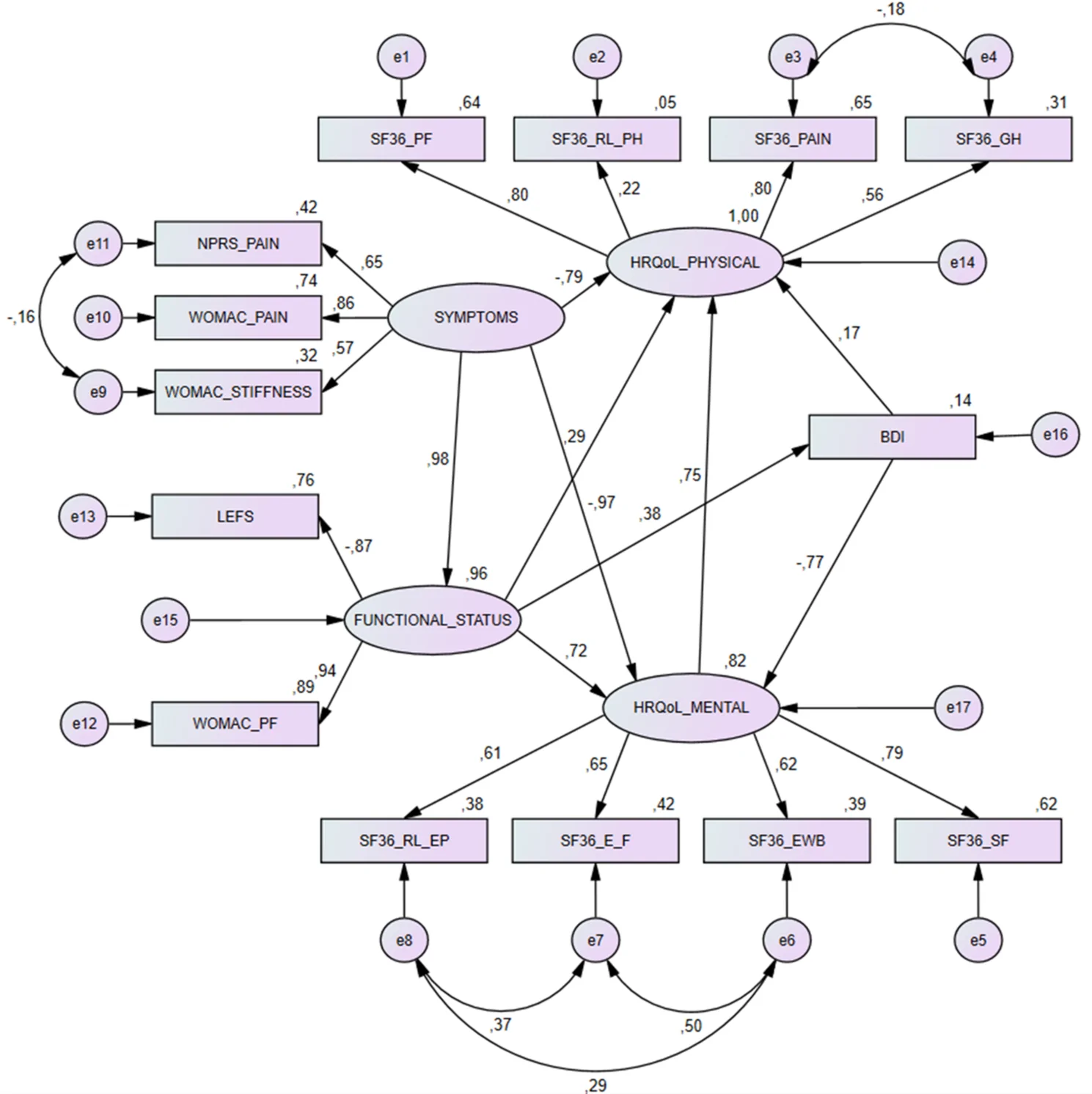
Structural equation model. BDI, Beck Depression Inventory; HRQoL_MENTAL, Health-Related Quality of Life Mental Component; HRQoL_PHYSICAL, Health-Related Quality of Life Physical Component; LEFS, Lower Extremity Functional Scale; NPRS_PAIN, Numeric Pain Rating Scale; SF36_E_F, Energy/Fatigue (Vitality); SF36_EWB, Emotional Well-Being; SF36_GH, General Health Perceptions; SF36_PAIN, Bodily Pain; SF36_PF, Physical Functioning; SF36_RL_EP, Role Limitations due to Emotional Problems; SF36_RL_PH, Role Limitations due to Physical Health; SF36_SF, Social Functioning; WOMAC_PAIN, Western Ontario and McMaster Universities Osteoarthritis Index - Pain; WOMAC_PF, Western Ontario and McMaster Universities Osteoarthritis Index - Physical Function; WOMAC_STIFFNESS, Western Ontario and McMaster Universities Osteoarthritis Index – Stiffness

Following the confirmation of model fit, the relationships among the variables were examined. The results of the hypothesis testing, as determined by path analysis, are presented in Fig. 2. These results include the standardised regression coefficient and the variance explained by the independent variables (R²).

### The role of symptoms in functional status

According to the SEM analysis, ‘Symptoms’ (pain and stiffness) were significantly and positively associated with patients’ functional status (standardized coefficient = 0.978; p < 0.001). In this model, ‘Symptoms’ emerged as the sole predictor of ‘Functional Status’ and explained 95.7% of the total variance in ‘Functional Status’ (R²=0.957).

### The role of functional status in depressive symptoms

Functional status was found to have a significant positive association with depressive symptoms (standardized coefficient = 0.377; *p* < 0.001). In this model, ‘Functional Status’ emerged as the sole predictor of ‘Depressive Symptoms’, explaining 14.2% of its total variance (R²=0.142).

### Health-related quality of life: mental component (HRQoL-M) predictors

The analysis of hypothesized variables for the Mental Component of HRQoL (HRQoL-M) revealed depression as the main predictor. Specifically, depressive symptoms demonstrated a significant and strong association with HRQoL-M (standardized coefficient = -0.773; *p* < 0.001), indicating that higher levels of depression correspond to lower HRQoL-M. However, neither symptoms (standardized coefficient = -0.973; *p* = 0.446) nor functional status (standardized coefficient = 0.724; *p* = 0.567) demonstrated a statistically significant direct pathway to HRQoL-M.

### Health-related quality of life: physical component (HRQoL-P) predictors

The analysis of hypothesized variables for the Physical Component of HRQoL (HRQoL-P) revealed HRQoL-M as a significant predictor (standardized coefficient = 0.746; *p* < 0.05). In contrast, symptoms (standardized coefficient = -0.791; *p* = 0.485), functional status (standardized coefficient = 0.288; *p* = 0.783), and depressive symptoms (standardized coefficient = 0.166; *p* = 0.560) did not show statistically significant direct pathways to HRQoL-P.

## Discussion

This study aimed to assess a theoretical model concerning the predictors of HRQoL and reveal the complex relationships between the variables. SEM was employed to examine the predictors of physical and mental HRQoL components in patients aged 40–65 with knee osteoarthritis. The results revealed that depressive symptoms emerged as a significant and strong factor associated with the mental component of HRQoL (HRQoL-M), whereas symptoms and functional status had no significant structural relationship. Furthermore, our results showed that HRQoL-M is not only an outcome variable, but also demonstrates an association with the physical component of HRQoL (HRQoL-P). Moreover, the findings indicate that symptoms demonstrate a significant and strong relationship with functional status, and that functional status shows a significant relationship with depressive symptoms. In the existing literature, studies analysing complex causal mechanisms related to predictors of HRQoL using SEM are limited. The simultaneous evaluation of these variables within a single model enabled the examination of the latent variables predicting HRQoL. This approach yielded not only the predictors of HRQoL but also the structural associations among these latent variables.

The results of our structural model showed that the symptoms factor has a significant and strong association with functional status. Remarkably, the symptoms alone explained 95.7% of the total variance in functional status. Consistent with our results, previous studies reported strong relationship between symptoms (pain and stiffness) and physical functional measures, characterizing it as a significant determinant of functional status [47–51]. In the literature, this relationship is explained by the “Avoidance Model”, which suggests that although restricting physical activity due to pain alleviates symptoms in the short term, it leads to decreased muscle strength and consequent functional impairment in the long term [52, 53]. Additionally, studies have shown that stiffness in patients reduces functional ability by limiting joint mobility [54].

KOA is a chronic condition characterised not only by symptoms or functional limitations, but also by the presence of accompanying depressive symptoms. As demonstrated in previous studies, the prevalence of depressive symptoms within this population ranges from 19.9% to 25.4% [12, 13]. The loss of physical function in patients with KOA has been demonstrated to be a critical factor in the onset of depressive symptoms. The findings of our study demonstrated that functional status are a significantly predictor of depressive symptoms. Consistent with our findings, Sugai et al. (2018) reported that difficulties in activities of daily living, particularly putting on/taking off socks and getting into/out of a car, triggered the onset of depressive symptoms in participants [10]. The same study found that individuals with high levels of functional impairment were 3.6 times more likely to present with depressive symptoms. In addition to these subjective complaints, objective physical performance tests also demonstrate a similar relationship. Iijima et al. (2018) found that depressive symptoms were more severe in patients who performed poorly on the Timed Up and Go (TUG) test and had greater functional limitations [11].

Depressive symptoms are considered one of the strong predictors of impaired HRQoL in individuals with knee osteoarthritis. It has been reported that patients with knee osteoarthritis exhibit depressive symptoms more frequently than healthy controls and, consequently, have a significantly lower quality of life [18, 32, 55]. Consistent with these studies, our results show that depressive symptoms are significantly associated with perceived HRQoL-M. Another finding from our study is that HRQoL-M demonstrates a significant relationship with HRQoL-P. When evaluated within the context of the overall model, the results reveal that functional status is explained by symptoms (pain and stiffness), and depression is explained by functional status. This mediating role has also been confirmed by other research [56]. Through a sequential pathway, the symptoms (pain, stiffness) and functional impairments are associated with higher depressive symptoms and lower HRQoL-M, thereby indirectly explaining the perceived HRQoL-P. This result suggests that the HRQoL-P is linked to mental processes associated with the severity of symptoms and changes in functional status.

The literature indicates that pain, a predominant symptom in knee osteoarthritis, is associated with both the physical and mental HRQoL of patients [18, 57–59]. Correspondingly, it has been documented that joint stiffness is also associated with quality of life [57]. However, in our structural model, no significant direct path was found from the ‘Symptoms’ latent variable to HRQoL. This result does not reject previous findings but rather may identify the underlying mediating mechanism. Park et al. (2021) also reported a significant direct path from pain to quality of life. The discrepancy in findings may be explained by the model structures; the previous study [56] only included pain intensity, whereas our ‘Symptoms’ latent variable encompasses both pain and stiffness. From a clinical perspective, pain alone has the potential to be directly associated with an individual’s perception of HRQoL. In contrast, stiffness predominantly serves as a factor associated with impaired mobility. In this context, stiffness is primarily associated with reduced functional capacity, rather than demonstrating a direct path to HRQoL. This relationship, explained by the deterioration of HRQoL associated with functional impairment, is both clinically plausible and consistent with the inherent characteristics of the variable.

A study found a direct effect from functional impairment, as assessed using the ‘Timed Up and Go’ test, on quality of life [60]. This finding suggests that reduced physical performance can directly impact quality of life. In contrast, in our study, functional status, as assessed using self-report measures, did not show a direct relationship with a lower quality of life. Instead, this relationship was primarily mediated by higher levels of depressive symptoms. This outcome suggests that ‘perceived’ functional loss may be associated with quality of life through mental processes.

From a clinical perspective, we recommend that treatment-resistant patients with KOA be managed using a holistic approach in which each parameter is addressed individually. Our findings indicate that in order to achieve a comprehensive improvement in quality of life, it is necessary to integrate symptom relief, the correction of functional impairment, and the treatment of accompanying depressive symptoms into the overall treatment plan.

Epidemiological data indicate that knee osteoarthritis becomes more prevalent after the age of 40, and that a substantial proportion of individuals with knee osteoarthritis are under 65 years of age [3, 61]. Accordingly, selecting individuals aged 40–65 for our study is consistent with the literature. Furthermore, selecting this age group provides a methodological advantage in terms of reducing the potential confounding effects of comorbidities seen in older age groups on the results. This demographic consistency with the literature supports the validity of the results in the target clinical population.

The present study is subject to certain limitations, including an imbalance in the gender distribution. While the higher proportion of females in our sample is an expected demographic finding consistent with the existing literature, a more gender-balanced sample would have allowed for the identification of potential gender-based differences in these structural relationships. Additionally, clinically important confounding factors such as age, sex, BMI, pain duration, and disease severity were not included as covariates in the structural equation model. Including these variables was found to cause over-parameterization, ultimately destabilizing the model fit. Therefore, future studies specifically designed to investigate the relationship of these confounding variables with the examined constructs are recommended.

Furthermore, a remarkably high structural relationship was observed between symptoms and functional status in our model. Prior to testing, statistical assumptions were verified, and the absence of multicollinearity was confirmed (Tolerance > 0.10, VIF < 10). Clinically, this strong association is highly anticipated, as functional limitations in patients with KOA are directly linked to symptom severity. However, this strong covariance might partially stem from the inherent nature of self-reported scales. Consequently, a limitation of the current study is that it relied on self-reported questionnaires to assess the patients’ perceived functional status. Although these tools provide valuable clinical insights, future studies would benefit from incorporating objective functional performance tests (e.g., TUG or gait assessments) to complement these subjective findings and provide a more comprehensive assessment.

A limitation of this study involves the recruitment process. Although initial study invitations were disseminated to healthcare professionals via social media, relying on this digital dissemination and subsequent snowball sampling among patients may have influenced the sample composition. This approach likely overrepresents individuals receiving care from digitally active professionals or patients who are highly connected within specific social networks. Consequently, patients outside these networks may be underrepresented, which could restrict the generalizability of our findings to the broader clinical KOA population.

## Conclusions

The results of this study demonstrate the structural pathways associated with HRQoL in patients with KOA. Furthermore, this study clarifies the structural relationships among these variables, thereby identifying the underlying mediating mechanisms. Through a sequential pathway, symptoms and functional impairments are linked to higher depressive symptoms and lower HRQoL-M. This subsequent lower level of HRQoL-M relates to the HRQoL-P. This result suggests that the HRQoL-P is connected to mental processes associated with the severity of symptoms and changes in functional status. From a clinical perspective, this mediating role should be considered when patients show no improvement in their quality of life despite conventional or surgical treatments. Ultimately, to achieve a comprehensive improvement in quality of life in patients with KOA, it is essential to integrate symptom relief, the management of functional impairment, and the treatment of accompanying depressive symptoms into a holistic treatment plan.

## Data Availability

The data that support this study cannot be publicly shared due to ethical or privacy reasons and may be shared upon reasonable request to the corresponding author if appropriate.

## Abbreviations

BDI: Beck Depression Inventory
HRQoL: Health-Related Quality of Life
HRQoL-M: Health-Related Quality of Life Mental Component
HRQoL-P: Health-Related Quality of Life Physical Component
KOA: Knee Osteoarthritis
LEFS: Lower Extremity Functional Scale
NPRS: Numeric Pain Rating Scale
SEM: Structural Equation Modeling
SF-36: RAND 36-Item Short Form Health Survey
WOMAC: Western Ontario and McMaster Universities Osteoarthritis Index
